# The Association Between Dietary Intake of Aromatic Amino Acids and Metabolic Syndrome

**DOI:** 10.1155/jnme/2102446

**Published:** 2025-11-12

**Authors:** Mahshid Rezaei, Somayeh Hosseinpour-Niazi, Neda Akhavan, Firoozeh Hosseini-Esfahani, Parvin Mirmiran, Fereidoun Azizi

**Affiliations:** ^1^Nutrition and Endocrine Research Center, Research Institute for Endocrine Disorders, Research Institute for Endocrine Sciences, Shahid Beheshti University of Medical Sciences, Tehran, Iran; ^2^Department of Kinesiology and Nutrition Sciences, School of Integrated Health Sciences, University of Nevada, Las Vegas 89154, Nevada, USA; ^3^Department of Clinical Nutrition and Dietetics, Faculty of Nutrition Sciences and Food Technology, National Nutrition and Food Technology Research Institute, Shahid Beheshti University of Medical Sciences, Tehran, Iran; ^4^Endocrine Research Center, Research Institute for Endocrine Disorders, Research Institute for Endocrine Sciences, Shahid Beheshti University of Medical Sciences, Tehran, Iran

**Keywords:** adults, aromatic amino acids, metabolic syndrome

## Abstract

**Background:**

The present study aimed to explore the association between dietary sources of aromatic amino acids (AAAs) from both animal and plant sources and the risk of developing metabolic syndrome (MetS).

**Objective:**

The primary goal of this research was to examine how the intake of AAAs correlates with the incidence of MetS.

**Methods:**

The current prospective observational study was conducted on collected data from 4920 adult individuals aged between 19 and 74 years, participants from Tehran Lipid and Glucose Study. Dietary data and usual intake of AAAs such as phenylalanine, tyrosine, and tryptophan were calculated using a validated semiquantitative food frequency questionnaire.

**Result:**

In this study, median intakes of AAAs were 5.9 g/d (IQR: 4.7–7.4 g/d), which corresponded to 2.8 g/d (2.2–3.4 g/d) of animal sources and 2.5 g/d (1.8–3.2 g/d) of plant sources. An increased intake of total AAAs (HR: 1.28; 95% CI: 1.05, 1.57) and AAAs from animal sources (HR: 1.30 and 95% CI: 1.03, 1.65) was positively linked to a higher risk of MetS, even after controlling for confounding variables. In different strata of BMI, the risk of MetS was positively associated with MetS only in obese subjects in the highest tertile of total AAAs intake (HR: 1.36 and 95% CI: 1.02–1.87) and for AAAs from animal sources (HR: 1.16 and 95% CI: 1.09–2.12). Nevertheless, none of the three BMI groups showed a statistically significant association between incident MetS and AAAs from plant sources. Also, no significant interactions were found between sex and AAAs intake and its constituents on MetS risk.

**Conclusion:**

The intake of AAAs, particularly from animal sources, was positively associated with the risk of MetS, especially in obese individuals. However, more studies are needed in this area.

## 1. Background

Metabolic syndrome (MetS) is characterized by the accumulation of abdominal adiposity, dyslipidemia (characterized by elevated triglycerides and low HDL cholesterol), as well as raised blood pressure and fasting plasma glucose levels [1]. Growing evidence indicates that all of these risk factors are linked to insulin resistance, which elevates the risk of cardiovascular disease and type 2 diabetes (T2DM) [2]. Prior investigations also reported that the MetS is very common, with an estimated prevalence of nearly 31.4% among the global general population of adults [3].

Both genetic and environmental factors play a significant role in the development of MetS. It is crucial to note that while the human genome has remained largely unchanged over the past decade, MetS prevalence has risen dramatically, underscoring the impact of environmental influences, especially dietary patterns [4]. Numerous studies have highlighted the potential benefits of plant-based dietary patterns, such as the Mediterranean and Dietary Approaches to Stop Hypertension (DASH) diets, on MetS and its associated components [5–7]. A recent meta-analysis showed that there was no significant association between adherence to plant-based foods and cardiometabolic risk factors when compared to a reduction in animal-based food intake [7–9]. Moreover, each additional serving of healthy plant-based foods was linked to a 4% lower risk of developing MetS [10].

There is a positive relationship between elevated levels of aromatic amino acids (AAAs) and the risk of T2DM and insulin resistance in both adults and adolescents [11–14]. Plant-based diets typically feature a lower intake of protein, as well as both essential and nonessential amino acids, which may enhance the benefits of these diets by leading to decreased serum amino acid levels [15–19]. An interaction has been observed between the Mediterranean diet, AAAs levels, which is associated with a lower risk of T2DM [15]. However, research on the effects of amino acids in chronic diseases like MetS remains limited and inconsistent [20, 21]. While longitudinal studies found a positive association between AAAs and insulin resistance, blood pressure, fasting glucose levels, and other components of MetS [12, 13, 22, 23], other studies reported no significant link with cardiometabolic risk factors [24–27].

## 2. Objectives

To address the previously mentioned gap in research, this population-based perspective study aimed to explore the relationship between the intake of AAAs over an 8.9-year follow-up period and the incidence of MetS. Additionally, we sought to investigate how the intake of animal and plant sources of these amino acids relates to the risk of developing MetS within the cohort of the Tehran Lipid and Glucose Study (TLGS).

## 3. Methods

### 3.1. Participants and Study Population

We conducted a prospective population-based study within the framework of the TLGS. This ongoing study aims to prevent noncommunicable diseases and encourage a healthy lifestyle. Our research included participants from three healthcare facilities in District 13, a region in Tehran, the capital of Iran. More detailed information about the TLGS methodology is available in previous publications [28, 29]. In total, 15,005 individuals aged 3 years and older from District 13 were selected through multistage cluster random sampling and have been followed over time. The study began in 1999, with participant follow-ups conducted every three years.

Participants were evaluated for sociodemographic and lifestyle factors, socioeconomic status (SES), medication usage, and medical history concerning cardiovascular risk factors. Additionally, anthropometric, biochemical assessments, and blood pressure measurements were conducted. This evaluation process began in 1999 and continued at 3-year intervals, with data collected during face-to-face visits by the local research team to update all prior information. The follow-up studies, designated as phases II, III, IV, V, and VI, were conducted prospectively from 2002 to 2005, 2006 to 2008, 2009 to 2011, 2012 to 2015, and 2016 to 2018, respectively.

**Figure 1:**
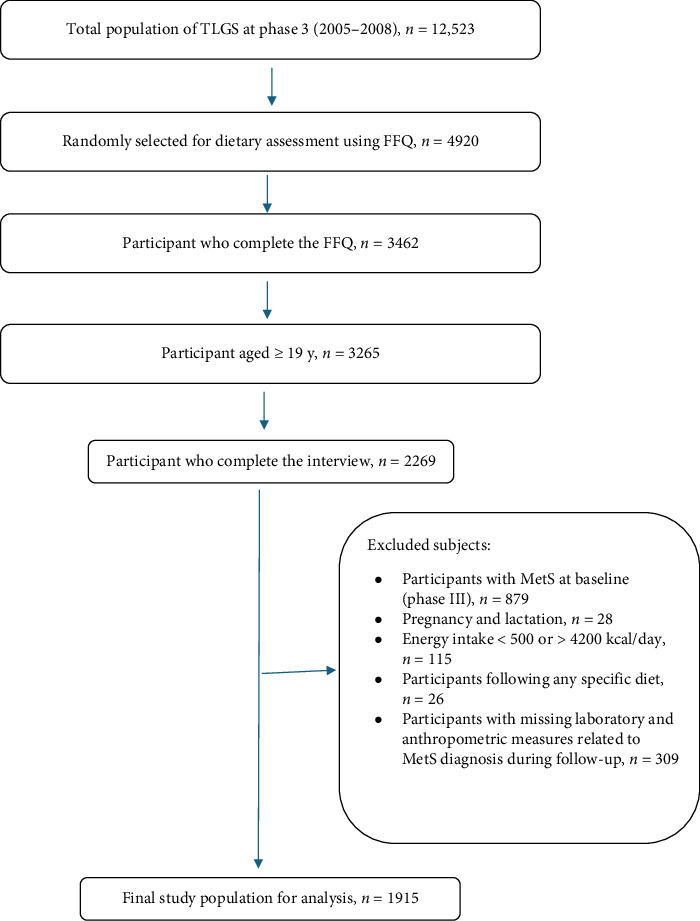
Flowchart of the study population, Tehran Lipid and Glucose Study (2006–2008 to 2016–2018).

During the third examination survey of the TLGS, conducted from 2006 to 2008, a total of 12,523 participants were assessed for medical history and underwent physical examinations. To obtain dietary data, we randomly selected a representative sample of 4920 participants based on age and gender, using a valid and reliable Food Frequency Questionnaire (FFQ) due to the cost, complexity, and time constraints involved in collecting dietary information from a large population. We compared the characteristics of these 4920 participants with those of the entire Phase III population to ensure they adequately represented the TLGS Phase III cohort. The data collected from these individuals were found to be similar to that of the overall Phase III population. [30]. Out of the 4920 selected participants, 3462 completed the questionnaire. Our focus was on 3265 adults aged 19–74 years who had complete demographic, anthropometric, biochemical, and dietary data. We excluded individuals based on the following criteria: those with MetS at baseline (*n* = 879), pregnant or lactating women at baseline or during follow-up (*n* = 28), individuals reporting daily energy intakes < 500 kcal and > 4200 kcal (*n* = 115), participants on specific diets or those who changed their diet at baseline or during follow-up (*n* = 26), and those with missing laboratory and anthropometric measures related to MetS diagnosis during follow-up (*n* = 309). Ultimately, the final analysis included data from 1915 participants collected between 2006 and 2018, resulting in a 66% response rate over the 8.9-year follow-up period (IQR: 7.98–9.69) (Figure 1).

This study protocol has been approved by the ethics committee of the Research Institute for Endocrine Sciences (RIES) at Shahid Beheshti University of Medical Sciences. All participants provided written informed consent.

### 3.2. Dietary Intake Assessment

Dietary assessment among TLGS participants was performed using a valid and reliable semiquantitative FFQ. [31]. Trained and experienced dietitians conducted face-to-face interviews to gather data on the frequency of intake for each food item over the previous year, recorded as daily, weekly, or monthly intake. Portion sizes reported in household measures were converted into grams. The energy and nutrient content were calculated using the Iranian food composition table (FCT). For micronutrients not available in the Iranian FCT, the USDA FCT was referenced, specifically the USDA National Nutrient Database for Standard Reference (https://www.ars.usda.gov/ba/bhnrc/ndl). Data on AAAs, including phenylalanine (Phe), tyrosine (Tyr), and tryptophan (Trp), were derived from a chemical analysis of the amino acid composition of over 5000 food items across all food groups.

In this study, given the significant impact of recent dietary intakes on the relationship between diet and chronic disease, we utilized an alternative approach based on the Han et al. formula [32]. This approach gives greater emphasis to recent dietary assessments, intending to reduce within-subject variability and provide a more concise evaluation of long-term diet [32]. Using this approach, the dietary intakes were calculated as the following: ((2016–2018 diet)/2 + (2012– 2015 diet)/4 + (2006–2008 diet)/8 + (2009–2011 diet)/8) [32]. Carried carried-forward method was used to impute the missing dietary intake values during follow-up.

There is a lack of information on the validity of the FFQ on AAAs, although intake of protein collected using the FFQ shows a valid estimate against multiple diet records. A good correlation coefficient was shown between FFQ and multiple 24 recalls (0.65 and 0.50 in males and females, respectively) and between two FFQs (0.79 and 0.69 in males and females, respectively) [31]. Moreover, there was reasonable reliability, validity, and stability of the dietary patterns, using data from the FFQ, over the 8 years [33].

Diet quality was evaluated using the Healthy Eating Index (HEI)-2020, which assesses how dietary intakes align with the Dietary Guidelines [34].

### 3.3. Physical Activity Assessment

A Modifiable Activity Questionnaire (MAQ) was used to estimate physical activity levels [35], which had previously been modified and validated among Iranians [36]. Participants were asked to indicate and acknowledge how often and how much time they engaged in light, moderate, and vigorous activities during the past 12 months, using a list of typical daily tasks. Physical activity levels were expressed as metabolic equivalent hours per week (MET-h/wk) [37].

#### 3.3.1. Clinical Measurements

Participants were interviewed by trained physicians and completed a questionnaire to provide demographic information such as age, gender, past medical history, and smoking habits. Weight was measured using a Seca digital weighing scale, designed for accuracy to the nearest 100 g (Seca 707; Seca Corporation, Hanover, Maryland; capacity range: 0.1–150 kg), while participants wore light clothing. Height was assessed utilizing a standing measuring meter with a precision of 0.5 cm, with participants positioned upright, barefoot, and ensuring that their shoulders were in a neutral alignment. Body mass index (BMI) was determined by dividing a person's weight in kilograms by the square of their height in meters. Waist circumference was assessed to the nearest 0.1 cm using an unstretched measuring tape at the level of the umbilicus, over light clothing, and without applying any pressure on the body. Blood pressure was recorded twice on the right arm after a minimum of 15 min of rest in a seated position, ensuring at least a 30-s interval between measurements. A mercury sphygmomanometer and the Korotkoff sound method were utilized, providing an accuracy of 2 mmHg. The final blood pressure reading was taken as the average of the two measurements. Systolic blood pressure was identified by the first audible sound, while diastolic blood pressure was noted at the beginning of the second sound.

### 3.4. Biochemical Measurement

Blood samples were collected in a sitting position between 7:00 and 9:00 a.m. after 12–14 h of overnight fasting and centrifuged within 30–45 min of collection. On the day of blood collection, all blood analyses were performed at the TLGS research laboratory. Samples were analyzed using the Selectra 2 autoanalyzer (Vital Scientific, Spankeren, the Netherlands). Enzymatic colorimetric kits were used to measure serum triglyceride levels (Pars Azmon Inc., Tehran, Iran). HDL-C was measured after Apo-lipoprotein B-containing lipoproteins were precipitated with phosphotungstic acid. The CVs for triglyceride and HDL-C were 0.5% and 2%, respectively. Fasting plasma glucose was measured using the enzymatic colorimetric method via glucose oxidase; both inter- and intra-assay coefficients of variation were lower than 2.3% at the baseline and follow-up phases.

### 3.5. Definition of MetS

As outlined in the Joint Interim Statement, a diagnosis of MetS requires the presence of three or more of the following criteria: [38]: (1) high blood glucose levels (fasting blood glucose ≥ 100 mg/dL or using antihyperglycemic medications), (2) elevated triglyceride level (≥ 150 mg/dL or treatment with antihypertriglyceridemia medications), (3) decreased HDL-C (< 50 mg/dL in women and < 40 mg/dL in men), (4) hypertension (≥ 130/85 mmHg or treatment with antihypertensive medications), and (5) an increased abdominal circumference (≥ 95 cm based on the population- and country-specific cutoff points for Iranian adults of both sexes) [39].

### 3.6. Statistical Analysis

For statistical analyses, SPSS, Version 15.0 (SPSS Inc., Chicago, IL, USA) was used. The residual method was used to calculate the energy-adjusted intakes of AAAs [40].

General linear models were used for the description of continuous variables and dietary intakes across tertiles of AAAs, and their constituents, as well as animal and plant sources of these amino acids. Description of categorical variables was done using the chi-square test. The data were presented as a mean (SD) or as a percentage. For the 8.9-year follow-up, an alternative approach was used for dietary variables to closely monitor long-term normal intakes and reduce in-person variance [41]. Using Kaplan–Meier survival analysis and Cox proportional hazards regression analyses, the hazard ratios (HRs) for the occurrence of MetS were estimated across tertiles of AAAs intake. Three models were constructed: Model 1 was crude. Model 2 was adjusted for age, gender, calorie intake, physical activity, occupation status, education status, HEI-2020, dietary fiber, and dietary cholesterol. Model 3 was, in addition, adjusted for BMI change (continuous variable) throughout follow-up. *p* values < 0.05 were considered statistically significant. Also, stratified analyses were conducted to assess effect modifications according to sex and BMI status (normal weight, overweight, and obese subjects).

We estimated the dose-response association between the intake of total AAAs and the risk of MetS using restricted cubic spline regression.

## 4. Results

During 8.9 years of follow-up, 591 new cases of MetS were documented. Forty percent were male. The mean age of the subjects was 36.5 ± 13.3 years, and the mean BMI was 25.6 ± 4.5 kg/m^2^. The median intake of AAA was 5.9 g/d (IQR: 4.7–7.4 g/d), which corresponded to 2.8 g/d (2.2–3.4 g/d) of animal sources and 2.5 g/d (1.8–3.2 g/d) of plant sources.

Subjects with higher intake of AAAs were younger, fewer smokers, more physically active, more educated, and more occupied. Intake of total energy, dietary fiber, dietary cholesterol, and dietary food groups is positively and intake of total dietary fat and protein is inversely associated with AAAs intake (Table 1).

The highest intake of AAAs was positively associated with the risk of MetS (HR: 1.26 and 95% CI: 1.04–1.54) when compared with the lowest tertile in Model 1. This association remained significant after adjustment for confounding factors in Model 2 (HR: 1.23 and 95% CI: 1.00–1.50) and Model 3 (HR: 1.28 and 95% CI: 1.05–1.57). Risk of MetS positively and significantly associated with intake of total AAA from an animal source (1.67 and 95% CI: 1.37–2.40) as well as intake of Trp (1.69 and 95% CI: 1.39–2.06), Phe (1.65 and 95% CI: 1.35–2.00), and Tyr (1.61 and CI: 1.32–1.96) from an animal source. Adjustment for confounding factors did not change these associations in Model 2 and Model 3. AAAs from plant sources showed no association with the MetS incidence in the crude model and after adjustment for confounding factors in Models 2 and 3 (Table 2).

**Figure 2:**
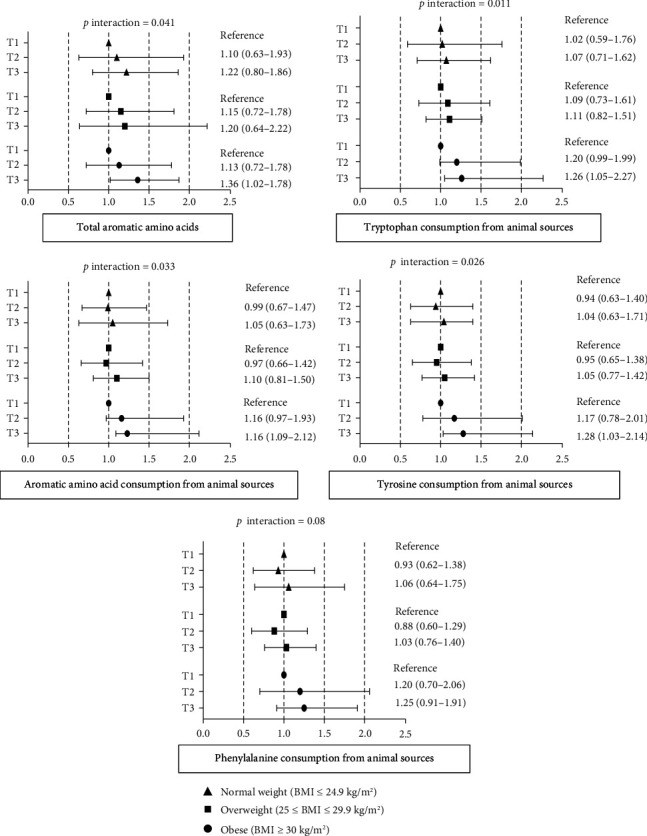
Multivariable hazard ratios (95% confidence interval) of the association between total aromatic amino acids (AAAs), as well as AAAs, tryptophan, tyrosine, and phenylalanine from animal sources and incident metabolic syndrome, stratified by BMI status. Data were adjusted for age, gender, calorie intake, physical activity, occupation status, education status, HEI-2020, dietary fiber, and dietary cholesterol.

In different strata of BMI, it was shown that the intake of total AAAs, as well as AAAs, Trp, and Tyr from animal sources, had different associations with MetS (Figure 2). The risk of MetS was positively associated with MetS only in obese subjects in the highest tertile of total AAAs intake (HR: 1.36 and 95% CI: 1.02–1.87) and for AAAs from animal sources (HR: 1.16 and 95% CI: 1.09–2.12), Trp from animal sources (HR: 1.26 and 95% CI: 1.05–2.27), and Tyr from an animal sources (HR: 1.28 and 95% CI: 1.03–2.14). Nevertheless, none of the three BMI groups showed a statistically significant association between incident MetS and AAAs and their constituents from plant sources. Also, no significant interactions were found between sex and AAAs intake and its constituents on MetS risk (Supporting Figure 1). Kaplan–Meier analysis illustrated a significant association between MetS and intake of total AAAs, as well as AAAs, Trp, Phe, and Tyr from animal sources (Supporting Figure 2).

**Figure 3:**
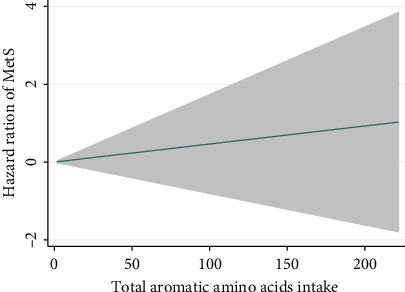
Dose-response association between aromatic amino acids intake and risk of MetS using restricted cubic splines. Data were adjusted for age, gender, calorie intake, physical activity, occupation status, education status, HEI-2020, dietary fiber, dietary cholesterol, and BMI change.

As illustrated in Figure 3, a linear relationship was observed between AAAs intakes concerning the risk of MetS (*p* value for nonlinearity of 0.359).

## 5. Discussion

In this 8.9 year follow-up study, we found that a diet high in total AAAs, as well as AAAs such as Phe, Trp, and Tyr from animal sources, is positively associated with the risk of MetS after adjusting for confounding factors. Additionally, our results indicated that BMI status influences these associations. Higher intake of AAAs and the aforementioned amino acids was positively linked to the risk of MetS, specifically among obese individuals.

Tyr, Phe, and Tyr are the three AAAs involved in the synthesis of a variety of proteins and secondary metabolites, which are involved in numerous anabolic pathways responsible for the production of many biological and neurological compounds that are essential for maintaining normal biological functions [32]. In the current study, we found that a high intake of AAAs is positively associated with the risk of MetS. Several studies have reported similar findings. In a prospective study involving 4754 nondiabetic individuals, after a 5-year follow-up, high levels of AAAs were associated with an increased risk of T2DM [42]. Additionally, longitudinal studies among obese children and young adults indicated a significant association between AAA levels and insulin resistance [22, 43]. A metabolomics investigation has also shown that elevated plasma concentrations of AAAs were related to the risk of T2DM [11]. However, contrary to these studies, some research has found no significant association with cardiometabolic risk factors [24–27]. The Ravansar noncommunicable disease cohort study did not identify a significant relationship between AAAs intake and the risk of T2DM. [25]. Furthermore, a case-control study involving adults with phenylketonuria (PKU) showed that, despite high Phe levels, there was no significant difference in HOMA-IR between PKU patients and healthy controls [24]. This inconsistency may be due to that the associations between insulin resistance and AAAs are modified by obesity [44]. In the current study, we found an interaction between AAAs intake and BMI status on the risk of MetS; this association was only observed among obese subjects. Our findings align with a prospective study that found a significant correlation between a higher serum Tyr concentration and increased HOMA-IR levels in overweight/obese participants, while no such association was found in those of underweight or normal weight [45]. Another prospective study indicated that the associations between higher serum levels of isoleucine, valine, and tyrosine with increased insulin resistance were significantly modified by obesity, being significant only in overweight and obese subjects [45]. The elevated circulating levels in these amino acids in individuals with obesity may results from excessive dietary intake or alterations in protein turnover or amino acid catabolism [45]. Increased AAAs levels might contribute to the onset of insulin resistance by disrupting the insulin signaling pathways [43]. Krebs et al. showed that plasma insulin concentrations were twice as high during amino acid infusion [46]. Similarly, Newgard et al. showed that rats fed with a high-fat diet supplemented with amino acids were more likely to develop insulin resistance related to obesity [47]. Moreover, changes in gut microbiota associated with obesity, particularly the reduction of Bacteroides involved in AAAs fermentation, may significantly contribute to rising AAAs levels, ultimately leading to insulin resistance [48].

In the current study, we observed that the intake of AAAs from animal sources is positively associated with the risk of MetS, whereas this association was not found for AAAs from plant sources. Although the type of protein plays an important role in determining AAAs levels, there is limited research on the metabolism of AAAs from animal and plant sources and their metabolites produced by microbial metabolism [49]. Plasma AAAs that are positively associated with diets high in animal protein can disrupt insulin function and lead to insulin resistance, thereby increasing the risk of MetS [50]. However, plant-based dietary patterns, such as Mediterranean and vegetarian diets, have been shown to significantly lower AAA levels [15–19]. Reduced AAA levels may contribute to a risk reduction in cardiometabolic factors. The PREDIMED trial indicated an interaction between the Mediterranean diet, AAAs, and Trp levels, which was associated with a reduced risk of T2DM [15]. Plant-based dietary patterns typically contain lower amounts of both essential and nonessential amino acids. The overall reduction in protein intake within these dietary patterns may enhance the benefits of plant-based diets by resulting in lower serum amino acid levels [51]. Additionally, consuming plant proteins and AAAs from legumes, nuts, and whole grains provides “microbiota-accessible carbohydrates” that upregulate the degradation of AAAs through gut microbial pathways [18, 49]. Therefore, adherence to plant-based dietary patterns has been linked to increased microbial diversity and higher production of beneficial AAA metabolites [52, 53]; however, findings are not entirely consistent. Consistent with our findings, some studies report no association between AAAs and insulin resistance, blood pressure, fasting glucose levels, and other components of MetS [24–27]. These discrepancies are often attributed to individual differences in diet quality, adherence levels, study duration, and variability in baseline microbiome composition.

The gut microbiota plays a crucial role in the development of MetS by metabolizing AAAs such as Trp, Phe, and Tyr into various bioactive molecules that influence host metabolic and immune pathways [54, 55]. In the conditions of microbial dysbiosis, which are often associated with insulin resistance, there is a significant reduction in healthy Trp-derived indole metabolites, such as indole-3-propionic acid (IPA), indole-3-acetic acid (IAA), and indole-3-aldehyde (IAld). These metabolites function as ligands for the aryl hydrocarbon receptor (AhR), prompting and maintaining mucosal immune homeostasis and the structural integrity of the gut barrier. A decrease in these compounds leads to increased gut permeability, metabolic endotoxemia, and systemic low-grade inflammation [56]. Simultaneously, high intake of AAAs, including Trp, results in a greater diversion of Try to the kynurenine pathway. This process, influenced by both host and microbial enzymatic activity, leads to the accumulation of proinflammatory metabolites derived from the abdominal fat, which are linked to insulin resistance and inflammation [54]. Additionally, Tyr is converted into p-cresol and its derivatives, which impair β-cell function and contribute to hepatic steatosis and systemic inflammation. Phe is metabolized to phenylacetylglutamine (PAGln), a compound associated with increased platelet hyper-reactivity [54]. Additionally, biogenic amines such as tyramine and phenylethylamine are produced through microbial decarboxylation of the AAAs, and these amines can disrupt the regulation of the sympathetic nervous system and appetite [57]. The metabolic dysregulation of features associated with MetS—including insulin resistance, dyslipidemia, visceral adiposity, and chronic inflammation—arises from the dysregulation of microbial metabolism of AAAs.

The current study has several strengths, including its population-based prospective design, long-term follow-up, the use of a validated FFQ, the use of an alternative approach to assessing dietary intake, and large numbers of samples. Furthermore, by conducting this study in the Middle East and North Africa, regions with different dietary patterns compared to Western and Eastern countries, we can enhance our knowledge of the relationship between protein and amino acid intake and chronic diseases such as MetS. There are certain limitations existing in our study that are worth mentioning. The observational nature of this study precludes assessment of cause and effect; however, the long follow-up period and comprehensive confounder adjustments help mitigate some of these concerns. Additionally, FFQ is not a robust tool to measure dietary intake as it mostly relies on the participants' memory. However, using the validation FFQ as well as alternative methods provides a more accurate evaluation of long-term dietary habits. The absence of sensitivity analyses to account for the measurement errors related to the FFQ is another of our study limitations.

## 6. Conclusion

In the current study, we found that high amounts of AAAs, especially from animal sources positively associated with the risk of MetS, particularly those with obesity. Nevertheless, additional prospective studies and clinical trials are needed to determine the effect of AAAs intake and its sources on the prevention and management of MetS.

## Figures and Tables

**Table 1:** Baseline characteristics and consumption of dietary variables across tertiles of total aromatic amino acids intake.

	T1	T2	T3	*p* value
Median intake (g/day)	4.3	5.9	8.5	
Range of intake (g/day)	≤ 5.1	5.2–6.8	≥ 6.9	
Participants (*n*)	639	638	638	
Age (y)	37.7 ± 0.5	36.4 ± 0.5	35.3 ± 0.5	0.005
Female *n* (%)	72.5	60.2	45.9	< 0.005
Physical activity (MET hr-week)	4.4 ± 0.3	4.8 ± 0.3	5.8 ± 0.3	< 0.005
Current smoking (%)	24.1	22.2	16.9	< 0.001
Academic degrees (%)	21.0	28.1	28.8	0.002
Occupational status, employed (%)	35.2	42.9	51.3	< 0.001
Family history of diabetes (%)	34.7	32.1	30.3	0.506
Family history of CVD events (%)	16.7	20.1	18.0	0.580
BMI at baseline (kg/m^2^)	25.5 ± 0.2	25.6 ± 0.2	25.6 ± 0.2	0.830
BMI change during follow-up (kg)	1.4 ± 0.1	1.5 ± 0.1	1.6 ± 0.1	< 0.001
Dietary variables				
Total energy (kcal/d)	2178 ± 48	2461 ± 48	2639 ± 49	< 0.001
Carbohydrate (% of total energy)	62.3 ± 0.5	61.5 ± 0.5	60.3 ± 0.5	0.010
Protein (% of total energy)	13.7 ± 0.3	14.6 ± 0.3	15.7 ± 0.3	< 0.001
Fat (% of total energy)	30.9 ± 0.2	29.8 ± 0.2	29.6 ± 0.2	< 0.001
SFA (% of total energy)	9.8 ± 0.1	9.8 ± 0.1	10.0 ± 0.1	0.442
MUFA (% of total energy)	10.7 ± 0.1	10.1 ± 0.1	9.9 ± 0.1	< 0.001
PUFA (% of total energy)	6.5 ± 0.1	6.0 ± 0.1	5.9 ± 0.1	< 0.000
Total fiber (g/d)	30.4 ± 0.6	41.4 ± 0.5	44.7 ± 0.6	< 0.001
Cholesterol (g/d)	159 ± 8	215 ± 8	323 ± 8	< 0.001
Vegetable (g/d)	242 ± 6	284 ± 6	337 ± 6	< 0.001
Fruit (g/d)	311 ± 12	388 ± 12	461 ± 12	< 0.001
Meat, processed meat, and organ meat (g/d)	19.0 ± 0.8	26.4 ± 0.8	38.8 ± 0.8	< 0.001
Poultry and fish (g/d)	24.9 ± 9.0	36.7 ± 9.0	73.6 ± 9.0	< 0.001
Whole grain (g/d)	91.4 ± 3.3	135 ± 3.3	199 ± 3.3	< 0.001
Refined grain (g/day)	266 ± 6	328 ± 6.0	402 ± 6.0	< 0.001
Legumes (g/d)	25.0 ± 1.1	36.4 ± 1.1	49.6 ± 1.1	< 0.001
Dairy products (g/d)	250 ± 7	376 ± 7	544 ± 7	< 0.001
HEI-2020	38.9 ± 6.8	42.8 ± 6.8	47.9 ± 6.8	< 0.001

*Note:* Dietary variables adjusted for age at baseline, gender, and total energy intake. Dietary data were computed using an alternative approach, due to the crucial impact of recent dietary intakes on the association between diet and chronic disease. Mean ± SE for continuous variable. p values determined using ANOVA for continuous variables and chi-square test for categorical variables.

Abbreviations: HEI-2020, Healthy Eating Index-2020; MUFA, monounsaturated fatty acids; PUFA, polyunsaturated fatty acids; SFA, saturated fatty acids.

**Table 2:** Multivariable adjusted hazard ratios (95% CI) for metabolic syndrome across tertiles of total aromatic amino acids (AAA) and its constituents.

Variables	Model 1				Model 2				Model 3			
	T1	T2	T3	*p* for trend^∗^	T1	T2	T3	*p* for trend^∗^	T1	T2	T3	*p* for trend^∗^
Total AAAs	1	1.16 (0.96–1.43)	1.26 (1.04–1.54)	0.02	1	1.23 (1.00–1.50)	1.30 (1.06–1.59)	0.001	1	1.15 (0.94–1.41)	**1.28 (1.05–1.57)**	**0.001**
Animal sources												
Total AAAs	1	1.12 (0.90–1.38)	1.67 (1.37–2.40)	0.001	1	1.03 (0.84–1.29)	1.41 (1.12–1.77)	0.01	1	1.02 (0.82–1.27)	**1.30 (1.03–1.65)**	**0.02**
Trp	1	1.09 (0.88–1.35)	1.69 (1.39–2.06)	0.001	1	1.01 (0.81–1.26)	1.37 (1.08–1.74)	0.001	1	1.03 (0.83–1.29)	**1.30 (1.02–1.66)**	**0.02**
Phe	1	1.14 (0.92–1.40)	1.65 (1.35–2.00)	0.001	1	1.04 (0.83–1.29)	1.32 (1.04–1.68)	0.03	1	1.07 (0.86–1.33)	**1.29 (1.01–1.64)**	0.05
Tyr	1	1.03 (0.83–1.30)	1.61 (1.32–1.96)	0.001	1	0.93 (0.75–1.15)	1.29 (1.02–1.63)	0.01	1	0.94 (0.76–1.17)	1.24 (0.98–1.59)	**0.04**
Plant sources												
Total AAAs	1	0.85 (0.70–1.04)	0.82 (0.67–1.00)	0.31	1	0.93 (0.74–1.18)	0.94 (0.70–1.26)	0.64	1	0.95 (0.70–1.09)	0.94 (0.70–1.26)	0.95
Trp	1	0.82 (0.68–1.00)	0.77 (0.65–0.95)	0.02	1	0.94 (0.73–1.21)	1.01 (0.72–1.41)	0.89	1	0.94 (0.73–1.21)	0.99 (0.71–1.37)	1.00
Phe	1	0.86 (0.71–1.04)	0.86 (0.71–1.04)	0.102	1	1.12 (0.87–1.43)	1.29 (0.94–1.75)	0.185	1	1.07 (0.83–1.36)	1.29 (0.83–1.36)	0.265
Tyr	1	0.82 (0.67–0.99)	0.82 (0.67–0.99)	0.02	1	0.99 (0.77–1.26)	1.07 (0.79–1.47)	0.74	1	0.94 (0.74–1.20)	1.02 (0.75–1.38)	0.95

*Note:* Model 1 was crude. Model 2 was adjusted for age, gender, calorie intake, BMI, physical activity, smoking status, occupation status, education status, dietary fiber, and dietary cholesterol, HEI-2020. Model 3 was additionally adjusted for BMI change (continuous variable) during follow-up. Trp, Tryptophan; Phe, phenylalanine; Tyr, Tyrosine. Bold values represent significant values.

Abbreviation: AAA, aromatic amino acids.

^∗^The median intake of each tertile category was assigned and then these quartile median variables were included as a continuous variable in Cox proportional hazards regression.

## Data Availability

The data that support the findings of this study are available on request from the corresponding author. The data are not publicly available due to privacy or ethical restrictions.

## Supporting Information

Supporting Information 1 Legend to Supporting Figure 1: Multivariable hazard ratios (95% confidence interval) of the association between total aromatic amino acids (AAAs), as well as AAAs, tryptophan, tyrosine, and phenylalanine from animal sources and incident metabolic syndrome, stratified by sex. Data were adjusted for age, gender, calorie intake, physical activity, occupation status, education status, HEI-2020, dietary fiber, and dietary cholesterol.

Supporting Information 2 Legend to Supporting Figure 2: Kaplan–Meier curves for the incidence of MetS outcomes by tertiles: (A) for total aromatic amino acid intake, (B) for aromatic amino acid intake derived from animal sources, (C) for phenylalanine derived from animal sources, D) for tryptophan derived from animal sources, and (E) for tyrosine derived from animal sources.
