# Supplementary material for: The Association Between Dietary Intake of Aromatic Amino Acids and Metabolic Syndrome
Source: J Nutr Metab. 2025 Nov 12;2025:2102446. doi: 10.1155/jnme/2102446 (PMC12629699; doi:10.1155/jnme/2102446)
Supplement: Supporting Information 1 — Legend to Supporting Figure 1: Multivariable hazard ratios (95% confidence interval) of the association between total aromatic amino acids (AAAs), as well as AAAs, tryptophan, tyrosine, and phenylalanine from animal sources and incident metabolic syndrome, stratified by sex. Data were adjusted for age, gender, calorie intake, physical activity, occupation status, education status, HEI-2020, dietary fiber, and dietary cholesterol. [file 2102446.f1.docx]

*P* interaction= 0.213


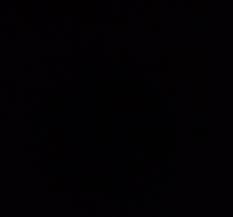
 Men


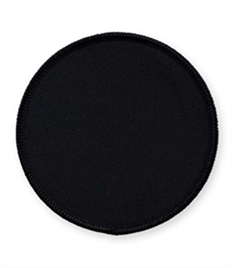
 Women

1.25 (0.81-1.93)

1.17 (0.75-1.82)

1.26 (0.89-1.79)

Reference

Reference

1.31 (0.98-1.75)

Total aromatic amino acids consumption

*P* interaction= 0.114

1.03 (0.76-1.39)

1.22 (0.93-1.62)

1.21 (0.82-1.79)

1.04 (0.70-1.53)

Reference

Reference

Aromatic amino acid consumption from animal sources

*P* interaction= 0.192

Tryptophan consumption from animal sources

1.16 (0.84-1.58)

Reference

1.15 (0.78-1.70)

Reference

1.23 (0.74-2.09)

1.33 (0.81-2.14)

*P* interaction= 0.148

Tyrosine consumption from animal sources

1.17 (0.88-1.56)

1.16 (0.80-1.69)

Reference

1.12 (0.76-1.63)

Reference

1.18 (0.86-1.61)

Phenylalanine consumption from animal sources

*P* interaction= 0.204

1.17 (0.88-1.56)

1.21 (0.83-1.77)

Reference

1.03 (0.71-1.51)

Reference

1.12 (0.81-1.53)
