# Supplementary material for: The Association Between Dietary Intake of Aromatic Amino Acids and Metabolic Syndrome
Source: J Nutr Metab. 2025 Nov 12;2025:2102446. doi: 10.1155/jnme/2102446 (PMC12629699; doi:10.1155/jnme/2102446)
Supplement: Supporting Information 2 — Legend to Supporting Figure 2: Kaplan–Meier curves for the incidence of MetS outcomes by tertiles: (A) for total aromatic amino acid intake, (B) for aromatic amino acid intake derived from animal sources, (C) for phenylalanine derived from animal sources, D) for tryptophan derived from animal sources, and (E) for tyrosine derived from animal sources. [file 2102446.f2.docx]

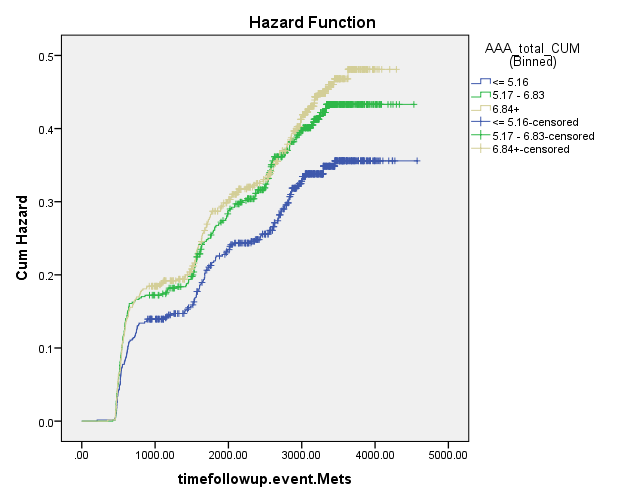


Log-rank test =0.025

A

Year of follow-up

Cumulative Hazard


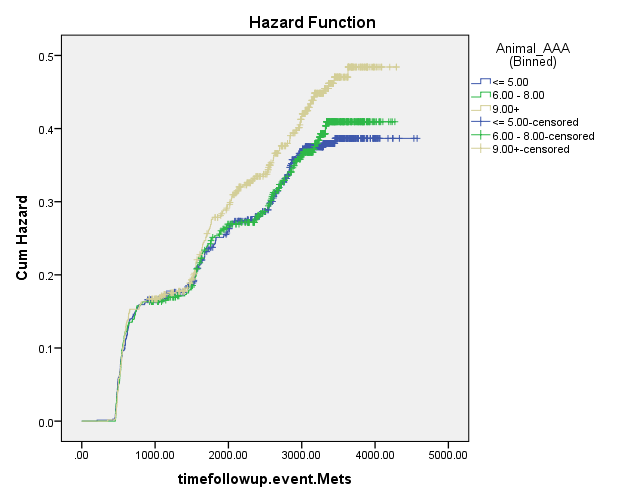


Log-rank test =0.014

B

Cumulative Hazard

Year of follow-up


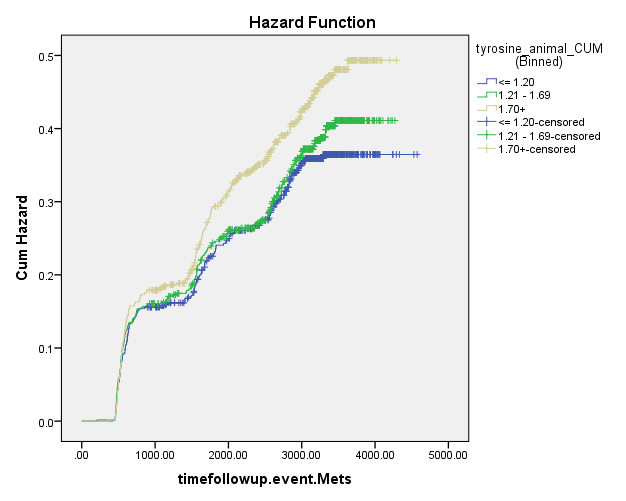


Log-rank test =0.050

C

Year of follow-up

Cumulative Hazard


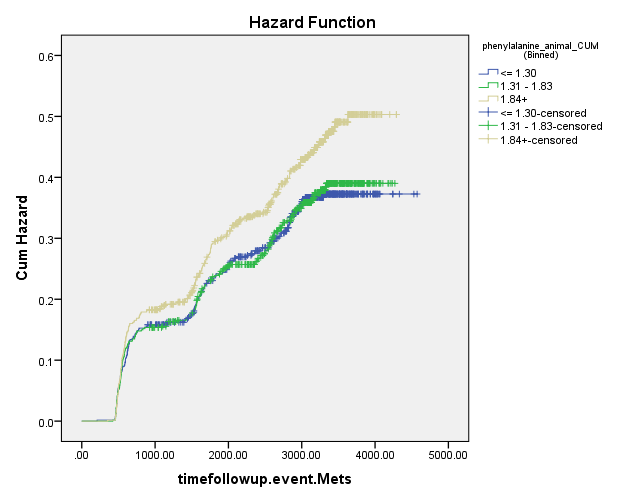


D

Log-rank test =0.037

Cumulative Hazard

Year of follow-up


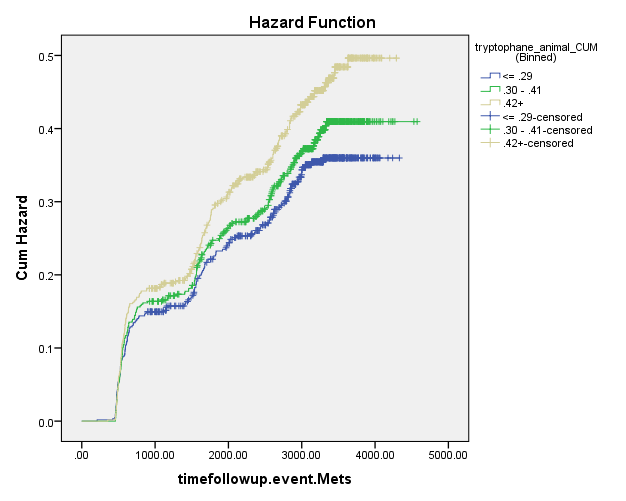


Log-rank test =0.033

E

Cumulative Hazard

Year of follow-up

Supplementary Figure 2. Kaplan-Meier curves for the incidence of MetS outcomes by tertiles: A) for total aromatic amino acid intake, B) for aromatic amino acid intake derived from animal sources, C) for phenylalanine derived from animal sources, D) for tryptophan derived from animal sources, E) for tyrosine derived from animal sources
